# One year of Hybrid Closed Loop on peritoneal dialysis: a case report

**DOI:** 10.1007/s00592-022-01880-5

**Published:** 2022-03-30

**Authors:** Antonio Rossi, Laura Montefusco, Ida Pastore, Maria Elena Lunati, Sabrina Argenti, Milena Muratori, Enrica Chebat, Gian Vincenzo Zuccotti, Maurizio Gallieni, Paolo Fiorina

**Affiliations:** 1 Division of Endocrinology, ASST Fatebenefratelli-Sacco, Milan, Italy; 2 Pediatric Clinical Research Center Romeo ed Enrica Invernizzi, Dept. Biomedical and Clinical Sciences “L. Sacco”, University of Milan and Pediatric Department, Buzzi Children’s Hospital, Milan, Italy; 3 Nephrology Division, Dept. Biomedical and Clinical Sciences “L. Sacco”, University of Milan, Milan, Italy; 4 International Center for T1D - Pediatric Clinical Research Center Romeo ed Enrica Invernizzi, Dept. Biomedical and Clinical Sciences “L. Sacco”, University of Milan, Milan, Italy; 5 Nephrology Division, Boston Children’s Hospital, Harvard Medical School, 300 Longwood Ave. Enders Building, Boston, MA 02115 USA

**Keywords:** Type 1 diabetes, Automated insulin delivery, Artificial pancreas, End stage renal failure, Peritoneal dialysis

## Abstract

**Background:**

Automated insulin delivery is a game changer for type 1 diabetes treatment.

**Objective:**

To describe the benefits of automated insulin delivery in a specific complex setting.

**Methods:**

We are herein presenting a case of a patient with type 1 diabetes, in which Hybrid Closed Loop (Medtronic Minimed 670G on Auto Mode) was used over a year during automated peritoneal dialysis. The patient was previously on insulin therapy with sensor augmented pump and we switched him to Hybrid Closed Loop shortly before the begin of dialysis.

**Results:**

Automated insulin delivery produced an increase of time in range (70-180 mg/dl) from 63% to 72%, after 3 months and to 74% after one year. Moreover, no hypoglycemia/hyperglycemia urgencies occurred overall during the year.

**Conclusions:**

The case detailed here is the first report of Hybrid Closed Loop in a patient on automated peritoneal dialysis and it shows an improvement of time in range with a satisfying safety profile in a fragile, aged patient.

## Introduction

Reaching the therapeutic objectives in type 1 diabetes is challenging as several patients are subjected to extreme glucose fluctuations. Glycemic targets in high risk patients, such as those with established atherosclerotic/cardiovascular disease or with chronic kidney disease, have been developed considering the avoidance of hypoglycemia as the main goal. At the same time, technology in diabetes is quickly changing the perspective of treatment. Algorithms for the automated insulin delivery are adding further benefits to continuous subcutaneous insulin infusion and real time continuous glucose monitoring. Full closed loops are still in study, conversely Hybrid Closed Loop and Advanced Hybrid Closed Loop, in which patients actively announce carbohydrates intake to bolus for the meal, is approved for type 1 diabetes treatment. Hybrid Closed Loops can improve HbA1c levels, the percentage of time spent in euglycemic range, the time below range and the quality of life in outpatients [1]. Medtronic MiniMed 670G system, MiniMed 780G, Control-IQ (Tandem Diabetes Care), DBLG1 (Diabeloop) and CamAPS FX (CamDiab) are closed loop systems with variable availability in different countries. Long-term benefits of these systems in higher-risk populations are still to be demonstrated. Interestingly, automated insulin delivery has been tested in patients with type 2 diabetes on artificial nutrition and admitted to in-hospital non-critical units [2]. One previous study described the automated insulin delivery approach in end stage renal disease, showing promising evidence of safety and efficacy in patients with type 2 diabetes receiving hemodialysis [3]. There is a lack of scientific evidence to support the use of a specific insulin regimen during peritoneal dialysis notwithstanding worst glycemic control has a negative impact on diabetic patients’ survival [4]. Peritoneal dialysis can have variable impact on glucose levels due to altered insulin metabolism, type of dialysis fluid employed and unpredictable intraperitoneal glucose absorption. Indeed, both hypoglycemia and hyperglycemia are common even in the presence of good HbA1c levels, and overnight glucose management can be particularly difficult. One prospective uncontrolled study shows good metabolic control with basal bolus insulin treatment and structured education [5] during peritoneal dialysis. There are no published data on continuous subcutaneous insulin infusion, sensor augmented pump or automated insulin delivery use. We are herein presenting a case of a patient with type 1 diabetes in which Hybrid Closed Loop (Medtronic Minimed 670G on Auto Mode) was used over one year during simultaneous automated peritoneal dialysis.

## Case description

A 77-year-old patient affected by type 1 diabetes since the age of 12 was admitted to our Diabetes Center three years ago. He developed over the years many diabetes complications, such as coronary heart disease, chronic kidney disease, proliferative diabetic retinopathy, cerebrovascular disease and diabetic polyneuropathy. He has been using insulin pump with glulisine analogue for 8 years, while in the last 4 years he used a sensor augmented pump with predictive low glucose suspend. The use of such device was excellently carried by the patient himself at the beginning and by the caregiver (wife) in the recent years, with rewarding consciousness and adherence to medical prescriptions. His target fasting glycemia was set to 140 mg/dl, with a mean time in range 70–80 mg/dl of 60% and no severe hypoglycemic events reported. Two months after the first presentation, he was admitted to the hospital for the worsening of kidney function and he underwent surgery to be prepared for peritoneal dialysis. At that time, glomerular filtration rate was 13 ml/min, HbA1c 63 mmol/mol (7.9%) and his body weight was of 65 kg with BMI of 21.5 kg/m2. Diabetes treatment was carefully reviewed by our team for the incoming intra-peritoneal glucose administration and for the lifestyle dramatic changes. Therefore, we proposed a switch to MiniMed 670G system, which consists in a 670G insulin pump, associated with a Guardian 3 Sensor for the continuous glucose monitor and a Contour Next Link blood glucose meter (Medtronic, Northridge, CA). On Auto Mode basal insulin delivery is adjusted according to glucose sensor every 5 min to reach the glucose target of 120 mg/dl. This is an Hybrid Closed Loop therefore unlike Advanced Closed Loop systems it doesn’t provide automated correction bolus. Both patient and caregiver underwent an intensive training with a 3 visits program over 2 weeks to implement the use of the new device, reassess carbohydrates count ability and review insulin/carbohydrates ratio. After 2 weeks of device use Auto Mode was activated. Six months after the first presentation automated peritoneal dialysis began in-hospital and went on at home with a schedule of 3 exchanges daily, administration of bicarbonate/lactate 1.36% glucose solution and of 7.5% icodextrin at nighttime and daytime, respectively. After few months, exchange program was changed with the removal of icodextrin, while bicarbonate/lactate solution exclusive was administered over night-time. CareLink^®^ Clinical Software reports were used to analyze metabolic outcomes.

## Results

**Fig. 1:**
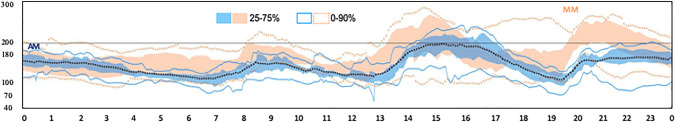
Patient’s report from Hybrid Closed Loop (Medtronic 670G system). Automated insulin delivery (Auto Mode, AM) on peritoneal dialysis vs sensor augmented pump use (Manual Mode, MM) before peritoneal dialysis: daily average sensor glucose with percentile distribution over 2 weeks. Filled shape 25–75%; open shape, 0–90%; black dotted line, average

Glycemic results with MiniMed 670G system before and after the begin of peritoneal dialysis are shown in Table 1 and Fig. 1. Time in range increased from 63% (before dialysis, on sensor augmented pump) to 72% (after 3 months of dialysis, on Hybrid Closed Loop). Time below range remained unchanged (0% of time) and coefficient of variation was never above the 36% threshold. After one year of Hybrid Closed Loop use, the time in range was 74%, coefficient of variation 24% and HbA1c was 55 mmol/mol (7.2%). Total insulin daily dose increased from 24 to 47 units per day without patient weight gain, mean daily Insulin to Carbohydrates ratio was increased from 1/26 to 1/21 and medium need of manual correction boluses on Hybrid Closed Loop increased from 0.2 to 0.8 times a day respect to dialysis begin.

**Table 1:** HbA1c, continuous glucose monitoring data, insulin dosing and pump settings over 1 year

	MM 4 months before APD begin	AM 3 months before APD	AM 4 weeks on APD	AM 3 months on APD	AM 1 year on APD
HbA1c (mmol/mol – %)	63 – 7.9	–	60 – 7.6	58 – 7.5	55 – 7.2
Mean glucose ± SD (mg/dl)	172 ± 48	172 ± 38	149 ± 35	159 ± 42	155 ± 38
Time In Range 70–180 mg/dl (%)	63	66	82	72	74
Time Below Range (%)	0	0	0	0	0
Coefficent of Variation (%)	27	22	23	26	24
GMI (%)	7.6	7.6	6.8	7.2	7.0
Auto Mode use (%)	0	96	97	97	98
Sensor Use (%)	88	98	99	98	86
Total Daily Insulin Dose (UI)	24	22	32	37	47
Mean Insulin to Carbohydrates ratio	26	24	22	22	21
Mean Daily correction bolus (n)	–	0.2	0.9	1.0	0.8

* AM* Auto Mode use;* MM* Manual Mode use;* APD* Automated Peritoneal Dialysis;* GMI* Glucose Management Indicator

## Discussion

It is important to determine if Hybrid Closed Loop can be offered to all patients with type 1 diabetes in the view of the potential improvement of glycometabolic control. We are presenting a clinical case of a fragile, aged and complicated patient with type 1 diabetes who used the Hybrid Closed Loop for over one year during simultaneous beginning of peritoneal dialysis. The patient maintained an enviable glucose control, reaching in some reports more than 80% of time in the range without excursions below hypoglycemia limits. He spent over 90% of time on Auto Mode, which is an essential requirement for such results. There was no need to change insulin to carbohydrate ratio for dinner, despite the glucose intraperitoneal infusion during nighttime. Indeed, we recorded an excellent performance of the automated insulin delivery during dialysis exchange. The total insulin daily dose increased of 40%, which is a much greater rise compared to what was detected on multiple dose insulin injection regimen [5], with percentage basal/bolus split reaching 70% in accordance with the intervention of auto mode corrections. No hyperglycemia neither hypoglycemia urgencies occurred despite glucose target was settled in a range that can be considered lower respect to accepted targets for such patients.

In conclusion, Hybrid Closed Loop appears to be safe and feasible for patients with type 1 diabetes on peritoneal dialysis as demonstrated by the improvement in time in range without increase of hypoglycemia risk shown in the present report. Intervention studies must be designed to better investigate automated insulin delivery effects during peritoneal dialysis.
